# Structural elucidation of Langat virus helicase unveils dual-target inhibition for broad-spectrum anti-flaviviruses strategy

**DOI:** 10.3389/fcimb.2025.1664344

**Published:** 2025-10-01

**Authors:** Ruixue Li, Zhen Han, Xiao He, Rongrong Zhong, Chen Chen

**Affiliations:** ^1^ Department of Biochemistry and Molecular Biology, School of Basic Medical Sciences, Tianjin Medical University, Tianjin, China; ^2^ School of Life Sciences, Tianjin University, Tianjin, China; ^3^ Department of Geriatrics, Tianjin Medical University General Hospital, Tianjin, China

**Keywords:** structure, helicase, Langat virus, Zafirlukast, ATP, RNA

## Abstract

**Introduction:**

Flaviviruses, such as dengue, Zika, and Langat virus (LGTV), pose significant global health threats, highlighting the urgent need for broad-spectrum antiviral therapies. This study focuses on the NS3 helicase of LGTV, a key enzyme in viral replication, aiming to elucidate its structure and identify high-potency inhibitors to facilitate rational drug design.

**Methods:**

The study employed an integrated approach: 1) Structural Biology: Determining the crystal structure of apo LGTV helicase. 2) Comparative Analysis: Aligning the structure with Zika (ZIKV) and dengue (DENV) virus helicases to assess conformational flexibility. 3) Virtual Screening: Screening 11,027 compounds to identify high-affinity inhibitors. 4) Molecular Modeling: Validating binding modes and stability via molecular docking and dynamics simulations. 5) Experimental Validation: Assessing pan-flavivirus affinity of lead compounds using Isothermal Titration Calorimetry (ITC).

**Results and discussion:**

The cloverleaf-shaped architecture of LGTV helicase was resolved, revealing conserved ATP- and RNA-binding sites. LGTV exhibited an intermediate conformational flexibility upon ATP binding compared to ZIKV and DENV helicases. Virtual screening identified six high-affinity hits, notably the repurposed drug Zafirlukast. Zafirlukast demonstrated a dual-targeting mechanism, engaging both the ATPase pocket and RNA-binding cleft. Molecular dynamics confirmed stable binding, and ITC validated its broad-spectrum affinity across flaviviruses. The study establishes the LGTV helicase as a robust model for antiviral development. Zafirlukast emerges as a promising prototype for dual-target inhibitors, capable of simultaneously obstructing ATP hydrolysis and RNA unwinding. These findings provide a strong foundation for designing novel broad-spectrum therapeutics against neurotropic flaviviruses.

## Highlights

- First high-resolution structure of Langat virus NS3 helicase reveals conserved ATP/RNA-binding pockets and cloverleaf-shaped architecture.
- Intermediate conformational flexibility in ATP-binding motif I distinguishes LGTV from ZIKV and DENV, informing species-specific drug design.
- Dual-target inhibitor Zafirlukast binds both ATPase and RNA sites, stabilizing the helicase with superior dynamics.
- Pan-flavivirus activity demonstrated for Zafirlukast via conserved residue interactions and broad-spectrum molecular docking.
- Dual inhibition strategy blocks ATP hydrolysis and RNA unwinding simultaneously, offering a resistance-resistant therapeutic approach.

## 1 Introduction

Flaviviruses are arthropod-borne pathogens responsible for severe human diseases including hemorrhagic fevers, encephalitis, and neurological disorders, transmitted to vertebrate hosts through blood-feeding vectors (Sampath and Padmanabhan, 2009). These viruses are categorized into three epidemiological groups based on transmission routes: mosquito-borne variants such as Dengue virus (DENV), Zika virus (ZIKV), Yellow fever virus (YFV), Japanese encephalitis virus (JEV), West Nile virus (WNV), Murray Valley encephalitis virus (MVEV), and Kokobera virus (KOKV) (Ngo et al., 2019; Fishburn et al., 2025); tick-borne species including Tick-borne encephalitis virus (TBEV), Powassan virus (POWV), and Langat virus (LGTV) (Lennemann and Coyne, 2017; Porter et al., 2025); and viruses with no known vectors exemplified by Modoc virus (MODV), Sokoluk virus (SOKV), Yokose virus (YOKV), and Jutiapa virus (JUTV) (Lin et al., 2019; Zhang et al., 2022). This classification reflects their distinct ecological transmission cycles while maintaining conserved biological features characteristic of the Flaviviridae family. These viruses are believed to be the most significant arboviruses based on the rapid spread combined with high burden of morbidity and mortality (Pierson and Diamond, 2020; Madere et al., 2025), making them a major public health concern in many regions around the world and emphasizing the need of developing antiviral agents against flaviviruses.

First isolated from *Ixodes granulatus* ticks in Malaysia and Thailand (Smith, 1956; Bancroft et al., 1976), LGTV is a tick-borne flavivirus characterized by a lipid-enveloped, single-stranded positive-sense RNA genome (Chevalier et al., 2025). The viral genome encodes a polyprotein post-translationally processed by viral and host proteases into structural (capsid, prM/M, E) and nonstructural (NS1–NS5) components (Polienko et al., 2025). Among these, NS3 serves as a multifunctional enzyme: its N-terminal domain exhibits serine protease activity (in complex with NS2B), while the C-terminal region harbors helicase and RNA 5′-triphosphatase activities critical for viral RNA replication (Schweitzer et al., 2025; Wullimann et al., 2025). The helicase domain adopts a canonical three-subdomain architecture that coordinates ATP hydrolysis with RNA unwinding through conserved structural motifs (Scaramozzino et al., 2002). Although historically considered non-pathogenic in humans and explored as a live-attenuated vaccine candidate against neurotropic flaviviruses, emerging evidence from animal models and human challenge studies reveals concerning neurotropism (Prikhod'ko et al., 2002). Experimental inoculation in mice, non-human primates, and human volunteers demonstrated neurological manifestations (meningoencephalitis, myelitis) and persistent neurological sequelae, suggesting undocumented risks of virulence reversion or immunopathological responses (Chen et al., 2020; Akaberi et al., 2021). These findings underscore the necessity to elucidate LGTV’s replication mechanisms—particularly NS3 helicase’s role in RNA remodeling—to inform both antiviral development and vaccine safety optimization.

The flavivirus NS3 helicase, a core component of viral replication, drives RNA double-strand unwinding via ATP hydrolysis and represents a critical target for antiviral drug development (Panayiotou et al., 2018; Yang et al., 2020). Current inhibitor strategies focus on two primary approaches: nucleoside analogues (suramin, NITD-731) that competitively block the ATP-binding pocket, and non-nucleoside inhibitors that employ allosteric mechanisms or obstruct RNA binding (Ivanova et al., 2025; Wang et al., 2025). Notable examples include the repurposed asthma drug zafirlukast, which binds the RNA channel of DENV/ZIKV helicases with broad-spectrum activity, and ivermectin, an antiparasitic agent that inhibits ZIKV/YFV by disrupting NS3-RNA interactions (LeCher et al., 2025; Yadav and Jena, 2025). Key challenges include viral mutational escape, off-target effects due to structural homology with human ATPases, and poor blood-brain barrier penetration for neurotropic viruses like WNV (Murtuja et al., 2025). To address these limitations, the development of dual-target inhibitors represents a strategic approach to improving therapeutic outcomes through synergistic efficacy enhancement and mitigation of resistance mechanisms (Qiao et al., 2025; Wei et al., 2025).

In this study, we resolved the structure of the Langat virus (LGTV) NS3 helicase and characterized its ATP- and RNA-binding sites. Structural analysis identified a conserved substrate-binding pocket at the interface of these sites, presenting a strategic target for antiviral drug design. Virtual screening of both active sites yielded six high-affinity compounds with strong binding potential to LGTV helicase. Molecular docking revealed that these compounds competitively occupy either the ATP-binding pocket or RNA-binding channel, providing a structural framework for rational drug optimization. Notably, Zafirlukast exhibited dual-target binding capability, engaging both the NTP-binding site and RNA-binding channel across multiple flavivirus helicases (including ZIKV, DENV, and TBEV), as confirmed by molecular docking and isothermal titration calorimetry (ITC). ITC quantification demonstrated robust binding affinities, validating its pan-flavivirus inhibitory potential. We propose a novel dual-target inhibition mechanism wherein Zafirlukast simultaneously obstructs ATP hydrolysis and RNA unwinding, effectively crippling helicase function. These findings establish a proof-of-concept for dual-target antiviral strategies, offering a promising avenue to circumvent resistance mechanisms in flavivirus therapeutics. The integrated structural, computational, and biophysical approach outlined here provides a blueprint for developing broad-spectrum inhibitors against emerging flaviviruses.

## 2 Methods

### 2.1 Expression and purification of Langat virus helicase

The Langat virus helicase gene (residues 177-621) was cloned into the pET-32M expression vector with EZ-HiFi Seamless Cloning Kit (GenStar, T196-20), incorporating an N-terminal rhinovirus 3C protease cleavage site. Recombinant protein expression was performed in *E. coli* BL21(DE3) cells (YEASEN, 11804ES80) grown in LB medium supplemented with 100 μg/mL ampicillin at 37°C. Protein production was induced with 500 μM IPTG at an OD_600_ of 0.6–0.8, followed by incubation at 18°C for 20 h. Cells were harvested by centrifugation (6,000 ×g, 15 min, 4°C) and resuspended in ice-cold PBS buffer (Life-iLab, AC08L011). After sonication (5 cycles of 30 sec pulse/60 sec rest at 40% amplitude), the lysate was clarified by centrifugation (8,000 ×g, 50 min, 4°C). The supernatant was loaded onto a Ni-NTA affinity column (Life-iLab, AP62L202) pre-equilibrated with lysis buffer. Weakly bound contaminants were removed using a high-stringency wash buffer (25 mM Na_2_HPO_4_, pH 8.0, 400 mM NaCl, 25 mM imidazole). The His-tagged fusion protein was eluted and treated with 3C protease (1:50 w/w) at 4°C for 13 h to remove the affinity tag. Cleaved protein was collected in the flow-through during a second Ni-NTA pass, concentrated via ultrafiltration (10 kDa cutoff), and further purified by sequential ion-exchange (HiTrap Q HP) and size-exclusion chromatography (Superdex 200 Increase 10/300 GL). Purified helicase was buffer-exchanged into storage buffer (10 mM Tris-HCl, pH 7.5, 140 mM NaCl, 5 mM DTT, 5% glycerol) and concentrated to working stocks of 5, 10, 15, and 20 mg/mL for downstream applications.

### 2.2 Crystallization and structure determination of Langat virus helicase

High-quality crystals of Langat virus helicase were obtained using the sitting-drop vapor diffusion method at 16°C over 48 5hours. Crystallization conditions comprised 0.2 M sodium malonate (pH 7.0) and 17% (w/v) polyethylene glycol 3350, with a protein concentration of 10 mg/mL. Prior to cryocooling, crystals were cryoprotected by brief soaking in mother liquor supplemented with 10% (v/v) ethylene glycol and flash-frozen in liquid nitrogen. X-ray diffraction data were collected at 100 K on beamline BL02U1 of the Shanghai Synchrotron Radiation Facility (SSRF). Data processing statistics are summarized in **Table 1**. The structure was solved by molecular replacement using an AlphaFold2-predicted model as the search template. Iterative model building and refinement were performed in Coot (version 4) and Phenix (version 1.21), respectively, with final validation through MolProbity. Structural visualization and figure preparation were conducted using PyMOL (v2.5.2) and ChimeraX (v1.5).

**Table 1:** Data collection and refinement of crystal structure of LGTV helicase.

Structure	Langat virus helicase
Data collection	
Wavelength (Å)	0.979183
Space group	*P* 4_1_ 2_1_ 2
Resolution range(Å)	49.13-2.48 (2.61-2.48)^*^
Unit cell a, b, c (Å)	68.018, 68.018, 213.125
α=β=γ (°)	90
Unique reflections	18766 (2670)
Completeness (%)	100 (100)
Redundancy	20.0
*I/σ(I)*	11.80 (2.60)
*R*_merge_(%)	14.7(131.2)
*CC_1/2_*	0.99 (0.89)
Refinement	
Resolution range(Å)	36.12-2.48 (2.57-2.48)^*^
Reflections used in refinement	18566 (1803)
Reflections used for *R*_free_	929 (96)
*R*_work_ (%)	23.9
*R*_free_ (%)	28.1
Protein residues	433
Water	13
Number of non-hydrogen	3461
Protein	3348
Solvent	13
RMSD values	
Bond lengths (Å)	0.002
Bond angles (°)	0.41
Ramachandran favored (%)	96.02
Ramachandran allowed (%)	3.98
Ramachandran outliers (%)	0.00

^*^Numbers in the brackets represent the highest resolution shell.

R_merge_ = Σ_hkl_Σ_j_|I_hkl_ − I_hkl_(J)|/Σ_hkl_Σ_j_|I_hkl_(J)|. I_hkl_(J) and I_hkl_ represent the jth and mean intensity of reflection hkl.

R_work_ = Σ_hkl_||F_obs_|-|F_calc_||/|F_obs_|. F_obs_ and F_calc_ represent the observed and calculated structure factors, respectively. R_free_ is the R factor calculated with 10 % of unique reflections as the test set.

The values are reported by PHENIX.

### 2.3 Molecular docking

The molecular docking workflow commenced with the preparation of inhibitor structures using Chem3D (23) (PerkinElmer), where ligands were energy-minimized and converted into PDBQT formats compatible with AutoDock (v.1.2.x) (Eberhardt et al., 2021). The Langat virus helicase structure served as the receptor, processed in AutoDock Tools 4.2 by removing crystallographic water molecules, adding polar hydrogens, and assigning Kollman charges. A docking grid spanning the ATP/RNA-binding sites (60 × 60 × 60 Å dimensions) was defined to encompass critical functional regions. Molecular docking was executed via AutoDock Vina 1.2.3 using the Lamarckian genetic algorithm with 20 independent runs per ligand and an exhaustiveness parameter of 50 to ensure robust conformational sampling. Post-docking analysis involved validation of binding poses in PyMOL 2.5 (https://pymol.org/), quantification of hydrogen bonds (≤3.5 Å) and hydrophobic interactions through LigPlot+ 2.2, and extraction of binding free energies (ΔG in kcal/mol) from Vina outputs. This integrated pipeline enabled systematic evaluation of ligand-receptor interactions while balancing computational efficiency and accuracy.

### 2.4 ATPase activity assay

ATPase activity was quantified using the ATPase/GTPase Activity Assay Kit (Sigma-Aldrich). Reactions were performed in 96-well plates by preincubating 10 μL Langat virus helicase (800 nM final concentration) in 20 μL assay buffer for 5 minutes at 25°C. Reactions were initiated by adding 10 μL ATP at varying concentrations (0–10 mM) and incubated for 50 minutes at 25°C. Enzymatic activity was terminated by adding 200 μL reagent buffer, followed by a 30-minute incubation at room temperature to allow colorimetric detection. Absorbance was measured at 620 nm to quantify inorganic phosphate release. Kinetic parameters (Michaelis constant, *K*_m_, and catalytic rate constant, k_cat_) were derived from a Lineweaver-Burk double-reciprocal plot generated using GraphPad Prism 10.1 software. Each sample was repeated for three times.

### 2.5 Isothermal titration calorimetry

Experiments were performed using a MicroCal iTC200 titration calorimeter (Malvern Instruments). ITC was used to evaluate the binding affinities between different flavivirus helicases and Zafirlukast. The compound was used at a concentration of 2 mM, while the helicase proteins were used at 200 μM. Zafirlukast was loaded into the instrument’s syringe, which was then immersed into the sample compartment containing the helicase protein. The titration protocol consisted of 19 injections: an initial 0.5 μl injection of 2 mM Zafirlukast followed by eighteen 2 μl injections, delivered at 90-second intervals. The experiment was conducted at 16°C with a stirring speed of 700 rpm. Following each injection, the microcalorimeter measured the heat changes associated with binding until thermodynamic equilibrium was reached. The binding isotherm was analyzed using the Origin 7.0 software package (OriginLab, Northampton, MA), which employs a single set of independent sites model to determine the thermodynamic binding constants and stoichiometry.

### 2.6 Molecular dynamics simulation

Molecular dynamics simulations were performed using GROMACS (2021) under periodic boundary conditions in the NPT ensemble, with the initial protein-ligand complex structure derived from molecular docking. The CHARMM36 force field was applied to the protein, ions, and TIP3P water molecules, while ligand parameters were generated using the CHARMM Generalized Force Field (CGenFF). The protein structure was processed via the pdb2gmx utility, with histidine residues protonated as HSD and termini appropriately capped. The complex was solvated in a cubic water box (93 × 93 × 93 Å^3^ containing ~22,000 water molecules) and neutralized with 0.15 M NaCl to achieve physiological ionic strength. Prior to production runs, the system underwent three-stage energy minimization using steepest descent (500,000 steps or until maximum force < 1000 kJ/mol·nm), progressively releasing restraints on water/ions, protein side chains, and ligands. This was followed by three-step NPT equilibration (50 ps each) employing the v-rescale thermostat (310 K, τ_p = 0.1 ps) and Berendsen barostat (1 bar, τ_p = 1.0 ps). The final 100 ns production simulation utilized a 2 fs timestep with LINCS bond constraints, PME electrostatics (1.4 nm cutoff), and maintained system conditions via v-rescale temperature coupling and Parrinello-Rahman pressure control (1 bar, τ_p = 2.0 ps). Water molecules were constrained using the SETTLE algorithm, while other hydrogen bonds were managed through LINCS.

### 2.7 Virtual screening

A compound library of 11,027 molecules was assembled from the APExBIO, TargetMol, and Selleck databases for virtual screening. The Langat virus helicase structure was prepared in the Maestro 11.9 platform using the OPLS3e force field through sequential steps: removal of water molecules and extraneous ions (with retention of Ni²^+^ ions in the active site), protonation at physiological pH, reconstruction of missing atoms and structural motifs, and energy minimization to optimize the protein conformation. Small-molecule ligands were processed via the LigPrep module with default parameters to generate ionization states and tautomers. The receptor grid for molecular docking was centered on the binding site of EGCG—a known positive-control ligand—defining a cubic docking box with 16 Å edge lengths encompassing the catalytic Zn²^+^-coordinated pocket. Prior to screening, the docking protocol was validated through ligand re-docking, achieving a root-mean-square deviation (RMSD) of <2.0 Å between the predicted and reference poses. The virtual screening cascade comprised an initial Standard Precision (SP) docking phase to prioritize high-affinity candidates, followed by refinement of top-ranked hits (binding energy <−7.0 kcal/mol) using Extra Precision (XP) docking. Selected complexes subsequently underwent binding free energy calculations via the MM/GBSA method and manual visual inspection to evaluate interaction patterns and pose rationality, ensuring robust identification of potential Langat virus helicase inhibitors.

## 3 Results

### 3.1 Overall structure of Langat virus helicase

**Figure 1:**
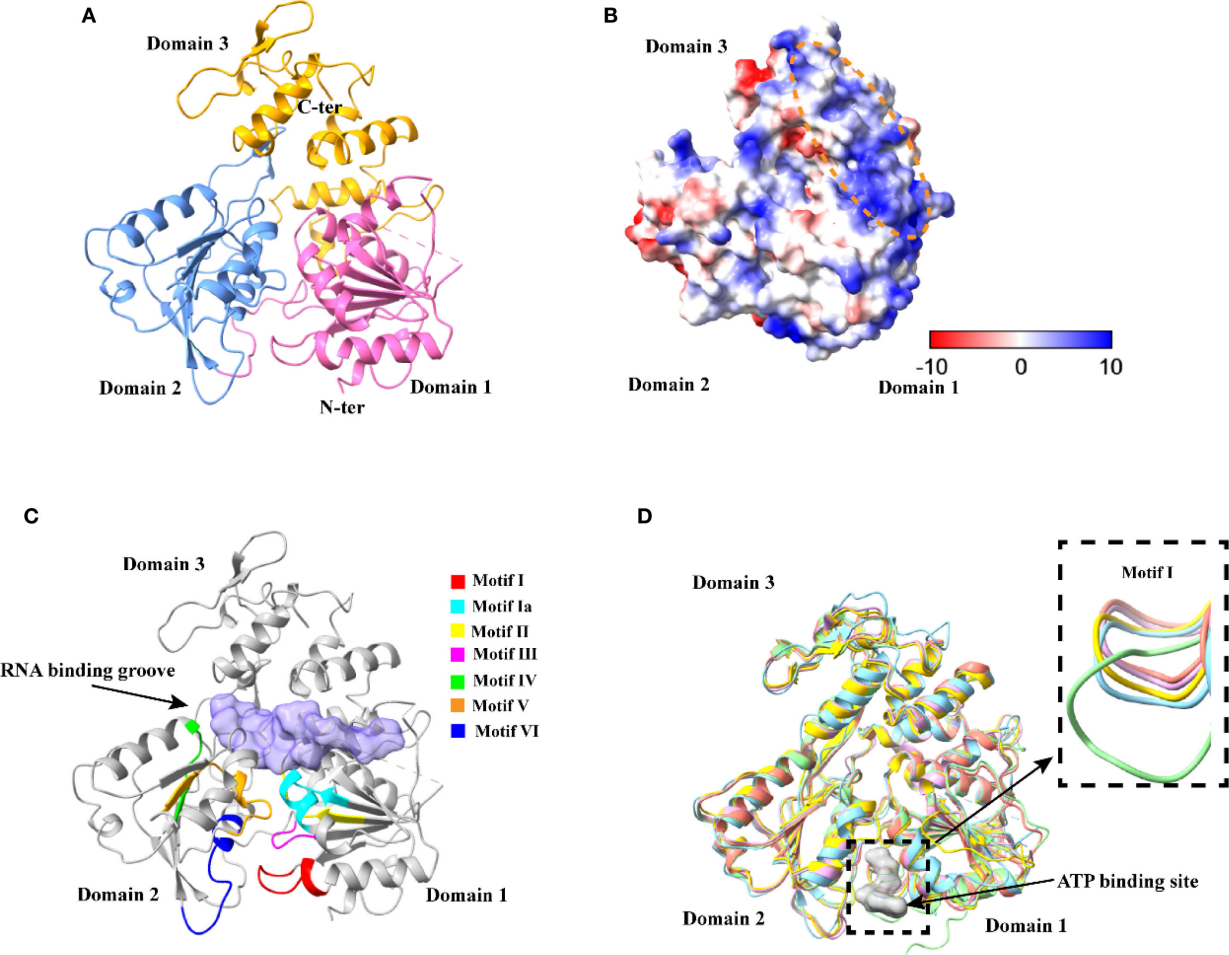
Overall structure of Langat virus helicase. **(A)** A ribbon model of Langat virus helicase crystal structure (PDB: 8YNJ, https://www.rcsb.org/structure/8YNJ). **(B)** The surface charge distribution and the RNA-binding site on the helicase of Langat virus. **(C)** Ribbon represents Langat virus helicase structure with RNA binding groove shown in violet, among which 7 conserved motifs are highlighted with different colors. **(D)** Comparison of P-loop of different flavivirus helicase structures, LGTV (PDB: 8YNJ), ZIKV-apo (PDB: 5JMT), ZIKV-ATP (PDB: 5GJC), DENV4-apo (PDB: 2JLQ) and DENV4-AMPPNP (PDB: 2JLR) are blue, yellow, pink, green and salmon, respectively.

The apo Langat virus helicase structure was determined in space group P4_1_2_1_2 at 2.48 Å resolution, with data collection and refinement statistics summarized in **Table 1**. The overall Langat virus helicase exhibits a cloverleaf-shaped architecture comprising three distinct domains (**Figure 1A**). Domain 1 features four α-helices and six β-strands containing conserved structural motifs I, Ia, II, and III. Domain 2 consists of six β-strands and four α-helices, with two helices extending into Domain 3 to form an interaction interface that incorporates conserved motifs IV, V, and VI (**Figure 1C**). Domain 3 is predominantly α-helical with two outward-protruding β-strands. These conserved domains collectively form critical functional sites for ATP binding/hydrolysis and RNA interaction essential for helicase activity. Further analysis of surface electrostatics of Langat virus helicase revealed a pronounced positive charge at the cleft formed by the separation of Domains 1/2 from Domain 3 (**Figure 1B**), a feature conserved in previous studies and consistent with its role in RNA binding, RNA can insert into the cleft formed by the separation of Domains 1/2 from Domain 3 (**Figure 1C**). Comparative structural analysis of the apo Langat virus helicase with related flavivirus helicases—including apo ZIKV helicase (PDB: 5JMT) (Tian et al., 2016a), ATP-bound ZIKV helicase (PDB: 5GJC) (Tian et al., 2016b), apo DENV4 helicase (PDB: 2JLQ) (Luo et al., 2008), and AMP-PNP-bound DENV4 helicase (PDB: 2JLR) (Luo et al., 2008)—revealed a high degree of overall structural conservation and conserved functional geometry: ATP binds within a groove at the interface of Domains 1 and 2. Among the seven conserved structural motifs, motif I exhibited the most pronounced positional shift upon ATP/AMP-PNP binding. While DENV4 helicase underwent substantial conformational changes (evidenced by large domain deflections) between its apo and ligand-bound states, ZIKV helicase displayed only minor structural rearrangements (**Supplementary Figure S1**). Intriguingly, the apo Langat virus helicase adopted an intermediate conformation for motif I, positioned between the apo states of ZIKV and DENV4 (**Figure 1D**). This suggests that Langat helicase’s ATP-induced conformational changes may similarly occupy an intermediate range among flaviviruses, highlighting divergent functional dynamics across viral lineages.

### 3.2 RNA binding groove of Langat virus helicase

**Figure 2:**
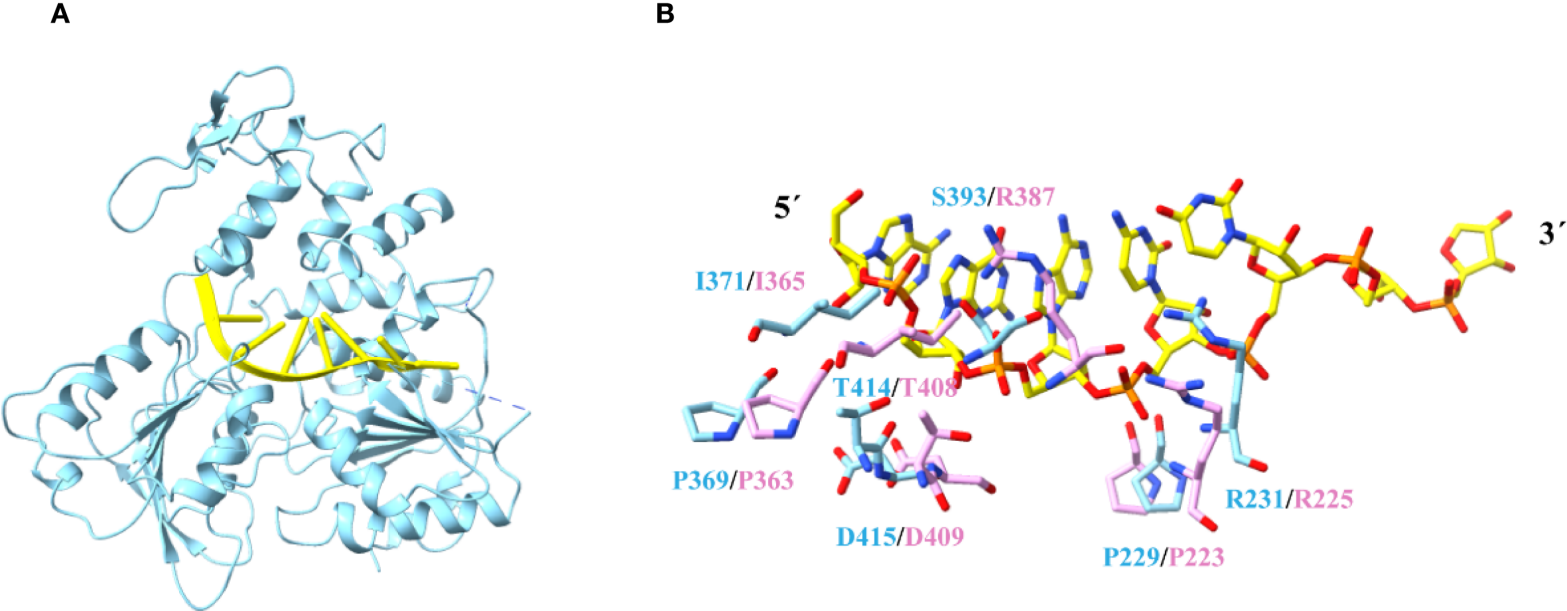
Structure of the helicase-RNA complex for Langat virus. **(A)** A model of a Langat virus helicase-RNA complex. The RNA was modeled into the Langat virus helicase structure by Alphafold3. The RNA is shown in yellow cartoon. **(B)** A comparison of residues which were predicted to contact RNA from Langat virus helicase (blue) and ZIKV helicase (pink). .

The RNA-binding groove, a surface pocket critical for mediating interactions between the NS3 helicase and NS5 polymerase, regulates viral replication. Structural analysis suggests that RNA engages the positively charged cleft of Langat virus helicase (**Figure 1B**), where ATP hydrolysis provides energy for duplex RNA unwinding, a mechanistic prerequisite for viral genome replication. We employed AlphaFold3 (Jumper et al., 2021; Tunyasuvunakool et al., 2021) to model the Langat virus helicase-RNA complex, enabling prediction of its RNA-binding interface (**Figure 2A**). The simulations revealed distinct binding preferences: the RNA 5′ terminus engages primarily with Domain 2, while the 3′ terminus associates with Domain 1. Key residues implicated in RNA binding include: Domain 1 (Motif Ia): P229 and R231, Domain 2 (Motif IV): P369 and I371, and Domain 2 (Motif V): T414 and D415 (**Figure 2B**). Notably, these residues are fully conserved across flaviviruses (**Supplementary Table S1**), supporting a universal RNA recognition mechanism essential for viral replication. This evolutionary conservation likely ensures functional stability within the flavivirus family.

### 3.3 NTPase activity pocket of the Langat virus helicase

**Figure 3:**
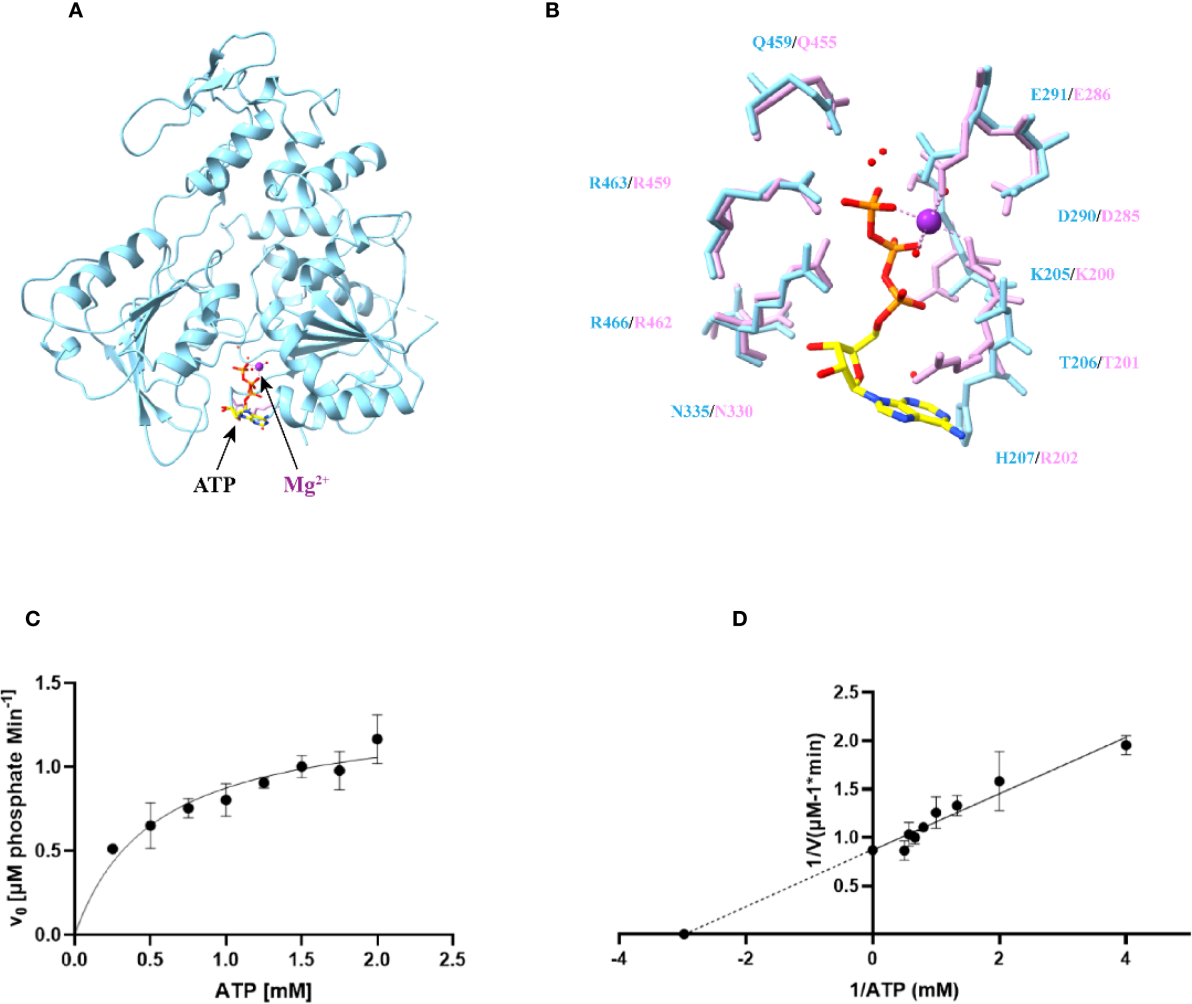
Structure of the NTP binding site of Langat virus helicase. **(A)** Predicted NTP binding site of the Langat virus helicase. **(B)** Comparison of residues predicted to bind ATP from Langat virus helicase (bule) and ZIKV helicase (orange). **(C, D)** The ATPase activities of the Langat virus helicase. The double-reciprocal plots were fitted according to the Michaelis-Menten equation. Each sample was repeated for three times.

The helicase is a critical enzyme in viral replication, coupling NTP hydrolysis to RNA unwinding during genome replication. Its NTPase activity not only powers RNA duplex separation but also modulates viral replication efficiency, thereby influencing the host innate immune response. Structural analysis of molecular docking by AlphaFold3 reveals ATP binds within a conserved groove at the interface of Domains 1 and 2, engaging structural motifs I/II (Domain 1) and motif VI (Domain 2) (**Figure 3A**). Comparative studies of LGTV helicase with ATP-bound ZIKV (PDB: 5GJC) and AMP-PNP-bound DENV4 (PDB: 2JLR) helicases demonstrate striking conservation of ATP-binding residues. Three critical basic residues—K205, R463, and R466 in LGTV (K200/R459/R462 in ZIKV; K199/R460/R463 in DENV4)—anchor the ATP triphosphate moiety (**Figure 3B**). Mechanistically, K205 stabilizes the catalytic site by coordinating the β/γ-phosphates. R463 and R466 form an arginine finger motif, positioning the γ/α-phosphates for hydrolysis. This conserved architecture underscores a universal NTPase mechanism among flaviviruses, essential for helicase function and viral fitness. In addition, we quantified the ATPase activity of Langat virus helicase using kinetic assays, revealing a Michaelis constant K_m_ of 0.336 ± 0.053 mM and a catalytic rate K_cat_ of 0.096 ± 0.004 s^−1^ (**Figures 3C, D**). Comparative analysis with other flavivirus helicases demonstrated distinct catalytic efficiency (K_cat_/K_m_) for Langat virus helicase, as evidenced by its significantly divergent ratio relative to homologs (**Supplementary Table S2**). This unique kinetic profile suggests evolutionary specialization in ATP utilization by Langat virus helicase.

### 3.4 Virtual screening-based discovery of dual-binding inhibitors targeting Langat virus helicase

**Figure 4:**
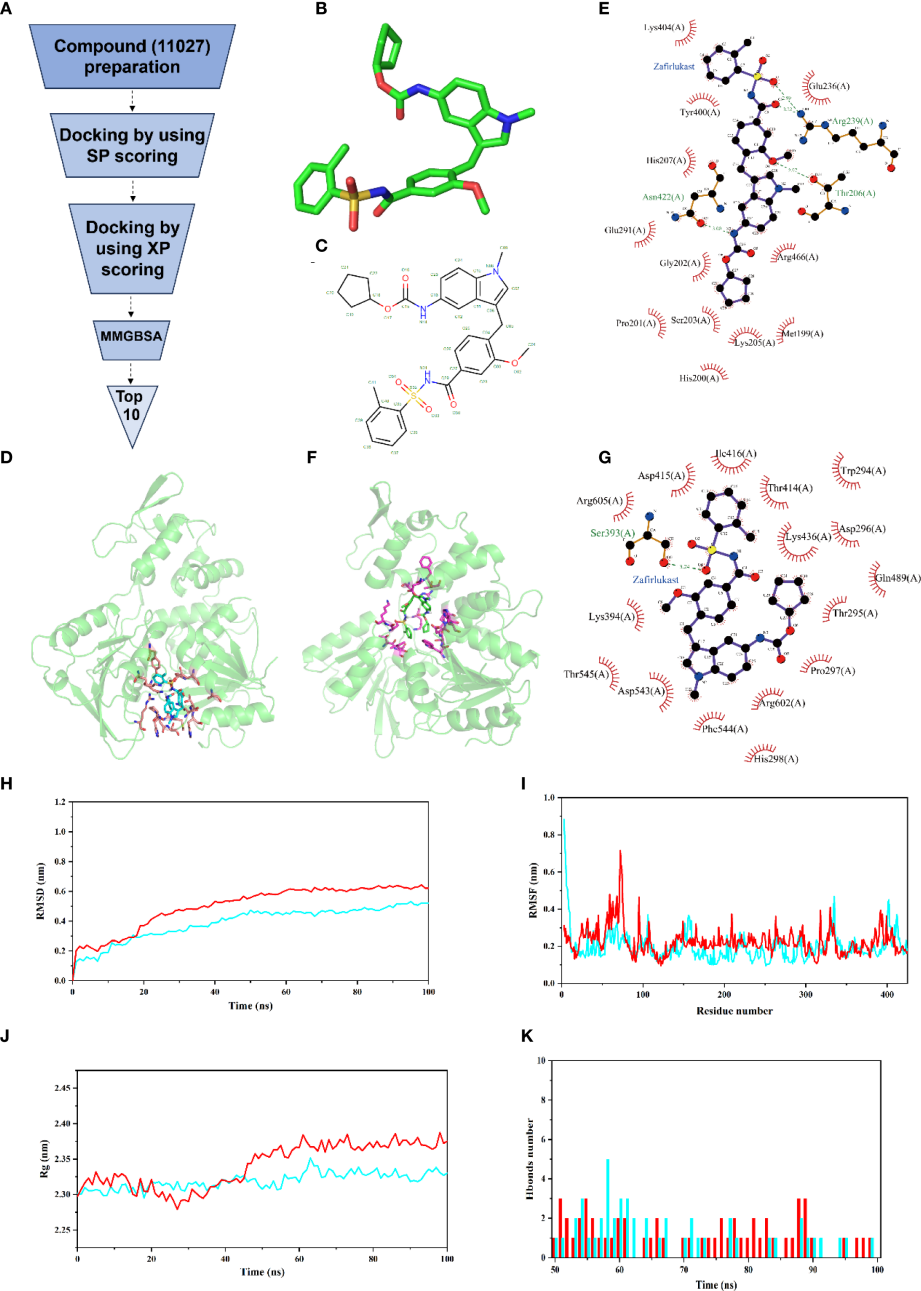
Discovery of inhibitors targeting Langat virus helicase. **(A)** Flowchart of virtual screening for Langat virus helicase inhibitors. **(B)** Three-dimensional structure diagram of Zafirlukast, carbon atoms are denoted in green, oxygen in red, nitrogen in blue, and sulfur in yellow. **(C)** Two-dimensional structure diagram of Zafirlukast, carbon atoms are denoted in black, oxygen in red, nitrogen in blue, and sulfur in yellow. **(D)** Predicted Zafirlukast binding to the Langat virus helicase NTP binding pocked, in this diagram, orange-red indicates amino acids in the ATP-binding pocket involved in interactions with the compound. The blue molecules of Zafirlukast correspond to its binding modes within the ATP-binding pocket. **(E)** Two-dimensional Zafirlukast- helicase NTP binding pocked interaction diagram of complex, carbon atoms are denoted in green, oxygen in red, nitrogen in blue, and sulfur in yellow. **(F)** Predicted Zafirlukast binding to the Langat virus helicase RNA binding groove, in this diagram, pink highlights amino acids within the RNA-binding groove that interact with Zafirlukast. The green and blue molecules of Zafirlukast correspond to its binding modes within the RNA-binding groove. **(G)** The two-dimensional Zafirlukast- helicase RNA binding groove interaction diagram of complex, carbon atoms are denoted in green, oxygen in red, nitrogen in blue, and sulfur in yellow. **(H, I)** RMSD and RMSF plot during molecular dynamics simulations of Langat helicase withZafirlukast (blue) and Bananin (red), RMSD trajectory indicated an average value below 2.0 Å, with the system reaching dynamic equilibrium after approximately 50 nanoseconds **(H)**. RMSF analysis further revealed smaller conformational fluctuations in the Zafirlukast–helicase complex and higher overall stability **(I)**. **(J, K)** The Rg and hydrogen bond number of Langat helicase withZafirlukast (blue) and Bananin (red), Hydrogen bond analysis showed that both inhibitors form at least three persistent hydrogen bonds with residues in the binding pocket **(J)**. Rg analysis demonstrated that Zafirlukast binding enhanced the structural stability of the protein **(K)**.

Using the LGTV helicase structure as a template, we performed virtual screening of the ApexBio compound library (11,027 entries, including FDA-approved drugs, natural products, and traditional Chinese medicines) targeting the NTP- and RNA-binding sites (**Figure 4A**). Six top candidates were prioritized based on docking scores and binding energies, with ten potential inhibitors listed in **Supplementary Table S3**. Among these ten, eight high-affinity compounds—Zafirlukast, Bananins, RK-33, Divanchrobactin, PF-03715455, SSYA10-001, Hypericin, and Sennoside A—exhibited robust interactions with LGTV helicase according to the docking scores, binding modes and key structural interactions (**Supplementary Table S2**). Notably, Bananins, a structurally unique antiviral agent featuring a trioxyadamantane-pyridoxal hybrid scaffold previously shown to inhibit SARS-CoV helicase ATPase/helicase activities (Wang et al., 2011; Hessel et al., 2023), demonstrated strong hydrophobic interactions and hydrogen bonds with Langat virus helicase. Additionally, RK-33, Divanchrobactin, PF-03715455, SSYA10-001, Hypericin, and Sennoside A formed stable interactions within the RNA-binding channel. Zafirlukast emerged as the top candidate with the highest binding affinity (ΔG < -13.31 kcal/mol), demonstrating dual-target potential as a repurposed leukotriene D4 antagonist originally developed for SARS-CoV-2 (Mehyar et al., 2021; Ghobain et al., 2022) (**Figures 4B, C**). In the NTP-binding pocket, Zafirlukast engaged in hydrophobic interactions with M199, H200, P201, G202, S203, K205, H207, E236, E291, Y400, K404, and R466, complemented by hydrogen bonds with T206, R239, and N422 (**Figures 4D, E**). Critically, residues K205, H207, E291, and R466 overlap with ATP-binding sites and are evolutionarily conserved across flaviviruses. When bound to the RNA channel, Zafirlukast exhibited comparable affinity (ΔG = -8.587 kcal/mol), forming hydrogen bonds with S393, and hydrophobic interactions with T295, P297, T414, D415, I416, K436 et al. (**Figures 4F, G**), residues that coincide with the conserved RNA-binding interface. This dual engagement of catalytic sites suggests enhanced efficacy compared to single-target inhibitors, while the RNA-binding groove’s critical role in replication and the structural congruence with ATP/RNA-bound states underscore its therapeutic potential. The evolutionary conservation of interacting residues further supports broad-spectrum applicability across flaviviruses.

To further investigate the interaction between the inhibitor and the Langat virus helicase, we performed a 100-nanosecond molecular dynamics simulation. The RMSD trajectory (**Figure 4H**) indicated an average value below 2.0 Å, with the system reaching dynamic equilibrium after approximately 50 nanoseconds. These results suggest that Zafirlukast binds more stably within the helicase active pocket than Bananin, forming a more stable complex. RMSF analysis further revealed smaller conformational fluctuations in the Zafirlukast–helicase complex and higher overall stability (**Figure 4I**). Hydrogen bond analysis showed that both inhibitors form at least three persistent hydrogen bonds with residues in the binding pocket (**Figure 4K**), which were critical for complex stability. Radius of gyration (Rg) analysis demonstrated that Zafirlukast binding enhanced the structural stability of the protein (**Figure 4J**). In summary, the simulations indicate that Zafirlukast engages the active site of the helicase via specific interactions, including a stable hydrogen-bond network, facilitating a tightly bound complex that likely underlies its inhibitory activity.

### 3.5 Conserved binding activity of Zafirlukast across flavivirus helicases

**Figure 5:**
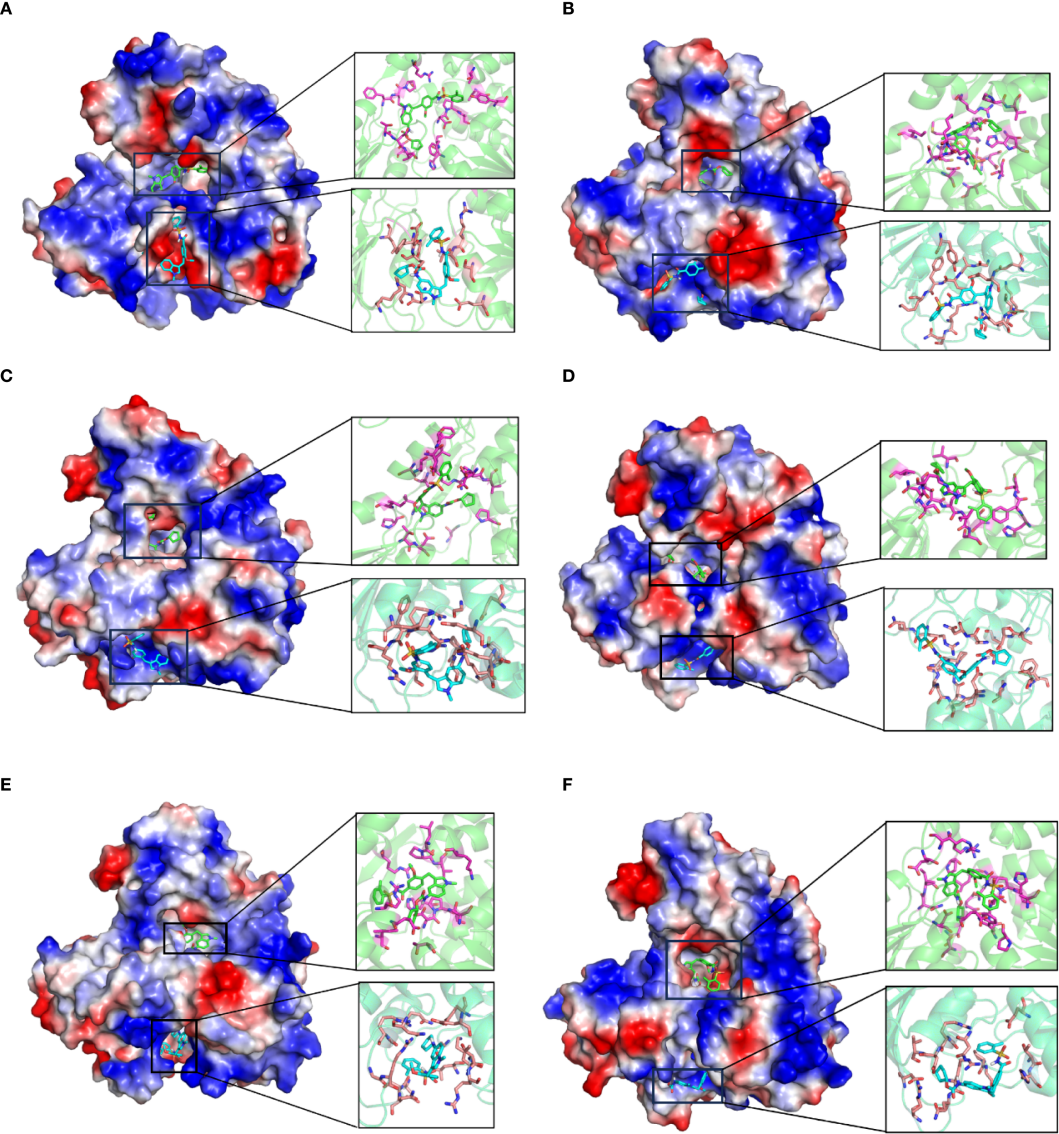
Conserved binding mode of Zafirlukast across flavivirus helicases. **(A–F)** Detailed view of Zafirlukast binding in both RNA binding groove (upper right) and NTP binding pocket (bottom right) of YFV **(A)**, DENV2 **(B)**, DENV4 **(C)**, JEV **(D)**, ZIKV **(E)** and TBEV **(F)** helicase. In panels **(A–F)**, electrostatic surface potential representations are provided for the helicase of each flavivirus, with blue indicating positive charges and red indicating negative charges. The figure on the right depicts detailed molecular interactions between Zafirlukast and various flavivirus helicases. In this diagram, green denotes the RNA-binding groove, blue represents the ATP-binding pocket, pink highlights amino acids within the RNA-binding groove that interact with Zafirlukast, and orange-red indicates amino acids in the ATP-binding pocket involved in interactions with the compound. The green and blue molecules of Zafirlukast correspond to its binding modes within the RNA-binding groove and the ATP-binding pocket, respectively.

**Figure 6:**
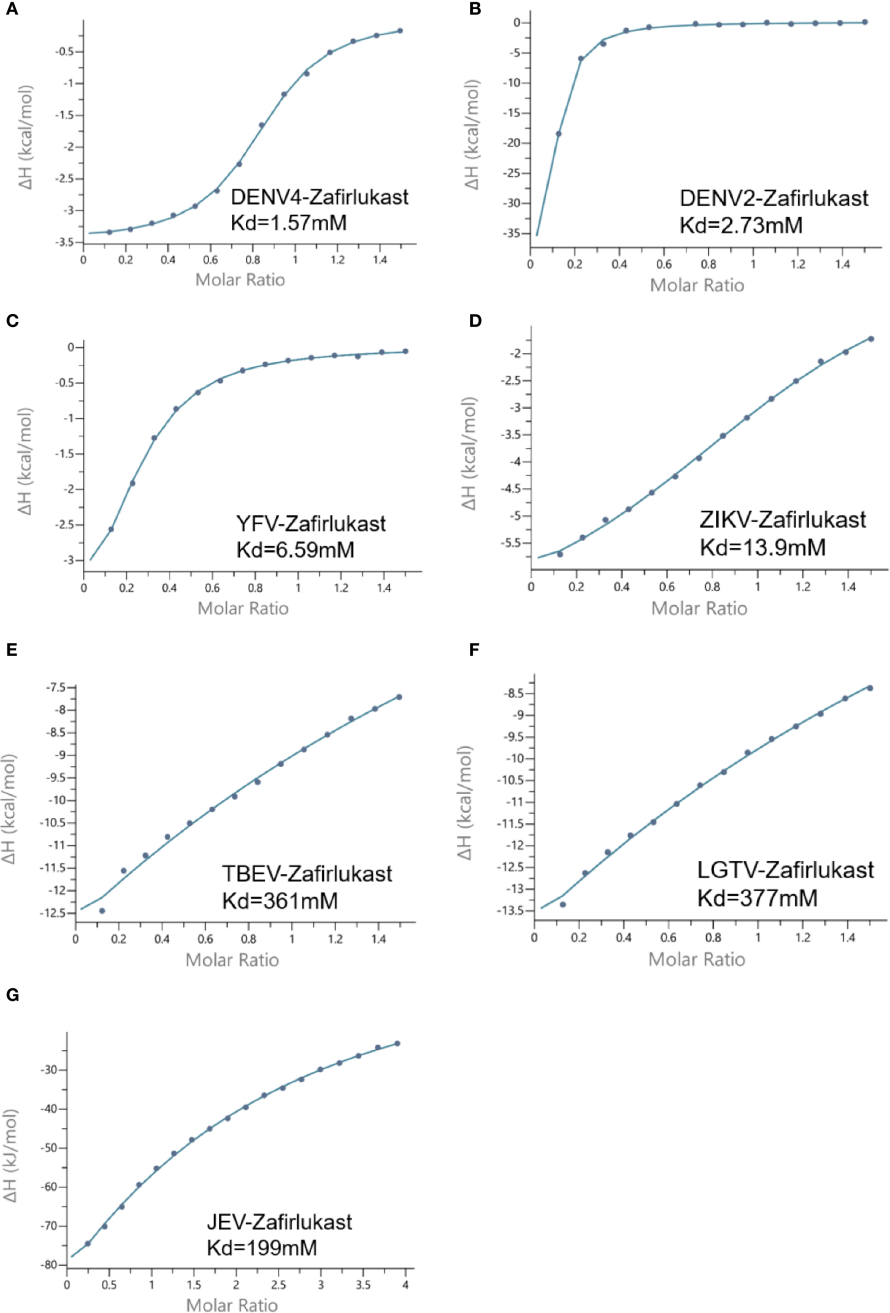
Binding activity of Zafirlukast across flavivirus helicases by ITC assays. **(A–F)** Binding affinity of Zafirlukast and DENV4 [**(A)** Kd=1.57mM], DENV2 [**(B)** Kd=2.73mM], YFV [**(C)** Kd=6.59mM], ZIKV [**(D)** Kd=13.9mM], TBEV [**(E)** Kd=361mM], LGTV [**(F)** Kd=377mM] and JEV [**(G)** Kd=199mM] helicase.

The high structural homology between Langat virus and other flaviviruses at both NTP- and RNA-binding sites suggests that inhibitors targeting Langat virus helicase may exhibit broad-spectrum activity against flaviviruses pathogens. Notably, Zafirlukast demonstrated strong binding affinity to both functional sites in Langat virus helicase, prompting investigation of its pan-flavivirus inhibitory potential. Molecular docking analyses with helicases from ZIKV, YFV, JEV, DENV2, DENV4, and TBEV revealed Zafirlukast’s consistent engagement of NTP-binding pockets and RNA-binding channels across all tested viruses, with binding energies below -7 kcal/mol indicating high affinity (**Table 2**). Particularly strong binding was observed with DENV4 (RNA channel: -10.982 kcal/mol; NTP pocket: -8.561 kcal/mol) and DENV2 (RNA channel: -10.146 kcal/mol), where RNA channel interactions universally surpassed NTP-binding affinity. Structural visualization using PyMOL 2.1 highlighted conserved interaction patterns: in Langat virus helicase, Zafirlukast formed hydrogen bonds and hydrophobic contacts with critical NTP-binding residues (K200, T201, R202, D285, E286, N330, Q455, R409, R462) within the conserved P-loop and motif VI, while also engaging RNA-channel residues likely to stabilize its position and obstruct substrate entry (**Figures 5A-F**; **Supplementary Figures S4**, **S5**). Isothermal titration calorimetry (ITC) confirmed robust binding across seven flavivirus helicases, with DENV4 exhibiting the strongest affinity (Kd = 1.57 μM) (**Figures 6A-G**). These findings collectively position Zafirlukast as a promising broad-spectrum inhibitor that exploits evolutionarily conserved structural features to disrupt helicase function through dual-site interference, providing a strategic template for anti-flavivirus drug development.

**Table 2:** Docking score of Zafirlukast to different flavivirus helicases.

Flavivirus	PDB number	Docking score (kcal/mol)	
		RNA binding groove	NTP binding pocket
LGTV	8YNJ	-8.587	-8.728
DENV4	2JLR	-10.982	-8.561
TBEV	7BLV	-9.067	-8.027
JEV	2Z83	-8.700	-8.566
YFV	1YKS	-9.540	-7.928
ZIKV	5JPS	-9.235	-7.921
DENV2	2BHR	-10.146	-7.731

All structural files referenced in this manuscript were retrieved from the RCSB Protein Data Bank (PDB) (https://www.rcsb.org/). Each PDB identifier consists of a unique alphanumeric code with four digits and capital letters. The docking score corresponds to the calculated free energy of binding between the inhibitor and the helicase of each flavivirus, expressed in kcal/mol.

## 4 Discussion

The LGTV NS3 helicase adopts a conserved three-domain architecture characteristic of flaviviruses helicases, featuring two critical functional pockets: an ATP-binding site positioned at the domain 1/2 interface and a surface-exposed RNA-binding cleft formed by the separation of domain 3. Comparative analysis of apo and ATP-bound states across flaviviruses [LGTV, DENV4 (Luo et al., 2008), ZIKV (Cao et al., 2016)] revealed virus-specific conformational dynamics in motif I upon ATP engagement. Notably, Langat virus helicase exhibited intermediate structural flexibility between the pronounced conformational shifts of DENV4 (Luo et al., 2008) and the relatively rigid ZIKV (Cao et al., 2016) helicase. This divergence in allosteric responses highlights the ATP-binding pocket as a hotspot for species-specific drug targeting. Molecular docking of two ATP-competitive inhibitors, Zafirlukast and Bananins, demonstrated their ability to engage conserved aromatic/hydrogen-bonding residues (e.g., K205, H207, E291, and R466) within the Langat virus helicase ATP pocket, rationalizing their inhibitory activity through steric hindrance of domain motions essential for ATP hydrolysis.

Potential molecular targets for developing broad-spectrum anti-flaviviral compounds encompass both viral proteins—such as the viral protease (Li et al., 2017; Shiryaev et al., 2017; Li et al., 2018) and polymerase (Ren et al., 2023; Ezzemani et al., 2024)—and host factors that are exploited during viral entry and replication, including α-glucosidase and proteins involved in nucleoside biosynthesis. Numerous compounds exhibiting broad-spectrum antiviral activity have already been discovered through target-specific or phenotypic screening approaches (Bonavia et al., 2011; Adalja and Inglesby, 2019; Karim et al., 2023). For additional candidates, broad-spectrum potential can be inferred based on their mechanism of action and the conservation of their molecular targets across flaviviruses (Geraghty et al., 2021; Hudson and Lockless, 2022). Bananins represents a class of structurally distinct antiviral compounds characterized by a trioxyadamantane moiety covalently linked to pyridoxal derivatives (Tanner et al., 2005; Wang et al., 2011). This compound is particularly notable for its dual inhibition of ATPase and helicase activities in SARS-CoV helicase (Tanner et al., 2005). Molecular docking analysis revealed that Bananins binds to LGTV NS3 helicase with a binding energy of -8.0 kcal/mol, forming hydrophobic interactions with residues P201, K205, V233, and G420, while hydrogen bonds were observed with G202, T206, E236, D290, and E291 (**Supplementary Figure S2**). Structural analysis of the ATP-binding site revealed preferential interactions with aromatic groups and hydrogen bond donors/acceptors. Consistent with its tunnel-shaped architecture, this site exhibits limited capacity to accommodate large molecular volumes. To elucidate the dynamic behavior of these interactions, 100-ns molecular dynamics (MD) simulations were performed on LGTV NS3 helicase complexes with Zafirlukast and Bananins. Root mean square deviation (RMSD) analysis demonstrated stable complex formation for both compounds, with global protein deviations remaining below 0.6 nm (**Figures 4H-K**). Notably, Zafirlukast exhibited enhanced stability compared to Bananins.

The RNA-binding cleft, characterized by its solvent accessibility and electropositive surface potential, displayed remarkable structural conservation across flavivirus helicases (Li et al., 2022; Wang et al., 2023). Virtual screening identified six RNA-binding candidates—Hypericin, Sennoside A, RK-33, SSYA10-001, PF-03715455, and Divanchrobactin—with Hypericin and Sennoside A exhibiting particularly strong computational affinity. An increasing number of studies have focused on the discovery of natural compounds with antiviral properties such as Hypericin (Cao et al., 2022; Mariangeli et al., 2024), Sennoside A (Esposito et al., 2016; Kumari et al., 2024) and Divanchrobactin (Chen et al., 2019). Interaction analysis revealed conserved recognition patterns: Hypericin formed π-stacking with W461 and hydrogen bonds with Q325/R458, while Sennoside A engaged T290 and K292 through its glycosyl moieties (**Supplementary Figure S2**). The conserved nature of these interactions across ZIKV (Cao et al., 2016), DENV (Luo et al., 2008), and WNV (Mastrangelo et al., 2007) helicases supports targeting this site for pan-flaviviruses inhibitor development. We evaluated multiple compounds interacting with LGTV NS3 helicase via molecular docking. Subsequent molecular dynamics simulations demonstrated that Hypericin and Sennoside A stabilized the protein structure with persistent hydrogen bonds, consistent with docking results (**Supplementary Figure S3**).

The dual conservation observed in both viral helicase functional sites (ATP/RNA pockets) present multifaceted targeting opportunities (Zhao et al., 2025). While ATP-site inhibitors like Bananins exploit virus-specific conformational plasticity for selective inhibition, RNA-binding molecules (Hypericin) mimetics leverage evolutionary constraints critical for helicase function across flaviviruses (Delcanale et al., 2022; Mohamed et al., 2022). These findings collectively validate helicase-targeted strategies ranging from narrow-spectrum allosteric modulators to broad-acting interfacial disruptors, providing a structural roadmap for developing next-generation antivirals against emerging flaviviruses.

Cysteinyl leukotriene receptor 1 antagonists (CysLTRAs), such as Montelukast, Zafirlukast, and Pulmonarust, are used in the management of asthma (Theron et al., 2014). Among these, Zafirlukast is an effective and well-tolerated agent recommended as preventive monotherapy for mild to moderate persistent asthma (Luginina et al., 2019). Emerging evidence supports its efficacy in combination with low-dose inhaled glucocorticoids. Although generally well-tolerated, Zafirlukast is associated with several safety concerns (Ye et al., 2025). Common adverse effects include mild headache, gastrointestinal disturbances, pharyngitis, and rhinitis; less frequently, rash and elevated aminotransferase levels may occur (Niet et al., 2024). Rare cases of vasomotor edema and neurogenic edema have been reported. At higher doses, it has been linked to an increased incidence of hepatocellular tumors, histiocytic sarcoma, and bladder cancer in preclinical models (Finnerty et al., 1992). Additionally, Zafirlukast exhibits off-target effects by inhibiting other pro-inflammatory pathways, including microsomal prostaglandin E2 synthase-1 (mPGES-1), 5-lipoxygenase (5-LO), cyclic adenosine monophosphate phosphodiesterase, and nuclear factor κB (Gunning et al., 2002; Lei et al., 2019). Given that flaviviruses cause systemic initial infection and primarily access the brain via the bloodstream, signs of barrier disruption—such as hemorrhagic lesions—often accompany neurotropic infections. These suggest that the virus may cross the brain endothelial barrier, leading to central nervous system invasion and neurological syndromes. Various viruses are capable of infecting and causing death in brain endothelial cells, which likely contributes to barrier breakdown and facilitates neuroinvasion (Hunter, 2022; Mielcarska and Rouse, 2025). Studies in rats using radiolabeled Zafirlukast show minimal distribution across the blood–brain barrier, highlighting a significant challenge for its repurposing or use in neurowasive infections (Zeng et al., 2020). Overcoming delivery obstacles remains critical for future therapeutic applications.

A limitation of this study is the absence of crystal structures for helicase–inhibitor complexes, as well as the lack of *in vitro* helicase inhibition assays and cellular and integrative validation of the antiviral activity of the screened inhibitors. Future studies should therefore focus on evaluating their efficacy, safety, and tolerability in relevant viral and animal models. While our computational and structural insights provide a valuable foundation for inhibitor development, several challenges must be addressed to translate these findings into clinical applications. Key limitations include the potential for off-target effect, suboptimal pharmacokinetic properties such as limited blood-brain barrier permeability for neurotropic viruses (Mielcarska and Rouse, 2025), and undefined regulatory pathways for repurposing existing drugs against new indications. Future studies will prioritize *in vitro* safety profiling against a broad panel of human kinases, comprehensive *in vivo* pharmacokinetic and toxicity studies in animal models, and the development of novel formulations or delivery systems to enhance bioavailability (Gunning et al., 2002). Furthermore, elucidating the precise regulatory requirements for drug repurposing will be essential for advancing promising candidates into clinical trials for flaviviral infections.

## 5 Conclusions

This study elucidates the high-resolution structure of Langat virus NS3 helicase, revealing conserved ATP- and RNA-binding pockets critical for its function in viral replication. The cloverleaf-shaped architecture and intermediate conformational flexibility of LGTV helicase distinguish it from related flaviviruses, providing insights into species-specific dynamics. Through virtual screening and molecular docking, we identified Zafirlukast as a dual-target inhibitor that simultaneously engages both ATPase and RNA-binding sites, achieving stable complex formation and broad-spectrum activity across flaviviruses. Molecular dynamics simulations and isothermal titration calorimetry validated its superior stabilization and pan-flavivirus affinity, highlighting a resistance-resistant mechanism by obstructing ATP hydrolysis and RNA unwinding. These findings establish LGTV helicase as a structural model for rational drug design and position Zafirlukast as a prototype for dual-target therapeutics, offering a strategic framework to combat neurotropic flaviviruses through conserved functional site targeting. This work underscores the potential of virtual screening-based approaches in developing next-generation antivirals with enhanced efficacy and reduced resistance risk.

## Data Availability

The original contributions presented in the study are included in the article/**Supplementary Material**. Further inquiries can be directed to the corresponding author.
