# Supplementary material for: Structural elucidation of Langat virus helicase unveils dual-target inhibition for broad-spectrum anti-flaviviruses strategy
Source: Front Cell Infect Microbiol. 2025 Oct 1;15:1664344. doi: 10.3389/fcimb.2025.1664344 (PMC12521441; doi:10.3389/fcimb.2025.1664344)
Supplement: Supplementary file 1 [file Table1.docx]

**Structural Elucidation of Langat Virus Helicase Unveils Dual-Target Inhibition for Broad-Spectrum Anti**-**Flaviviruses Strategy**

Ruixue Li^a #^, Zhen Han^b #^, Xiao He^a #^, Rongrong Zhong^c^ , Chen Chen^a^ *

^a^ Department of Biochemistry and Molecular Biology, School of Basic Medical Sciences, Tianjin Medical University, Tianjin, 300070, China

^b^ School of Life Sciences, Tianjin University, Tianjin, 300072, China

^c^ Department of Geriatrics, Tianjin Medical University General Hospital, Tianjin 300052, China

^#^These authors contributed equally to this work.

*Corresponding authors.

E-mail addresses: chench@tmu.edu.cn (Chen Chen).

**Contents**

**Figures**

**Fig. 1.** Overall structure of Langat virus helicase.

**Fig. 2.** Structure of the helicase-RNA complex for Langat virus.

**Fig. 3.**Structure of the NTP binding site of Langat virus helicase.

**Fig. 4.**Discovery of inhibitors targeting Langat virus helicase.

**Fig. 5.**Conserved binding mode of Zafirlukast across flavivirus helicases.

**Fig. 6.**Binding activity of Zafirlukast across flavivirus helicases.

**Fig. S1.** Structure-based alignment of Flaviviridae helicases.

**Fig. S2.**Conserved binding mode of different inhibitors across Langat virus helicase.

**Fig. S3.**Discovery of inhibitors targeting Langat virus helicase.

**Fig. S4.**Conserved binding mode of Zafirlukast across flavivirus helicases.

**Fig. S5.**Conserved binding mode of Zafirlukast across flavivirus helicases.

**Tables**

**Table 1.** Data collection and refinement statistics.

**Table 2.** Docking score of Zafirlukast to different flavivirus helicases.

**Table S1.** Comparison of residues contacting with RNA from Langat virus, ZIKV and DENV4 helicase.

**Table S2.** The ATPase activity comparison of different flaviviruses helicase.

**Table S3.** Candidate inhibitors of LGTV helicase by virtual screening.


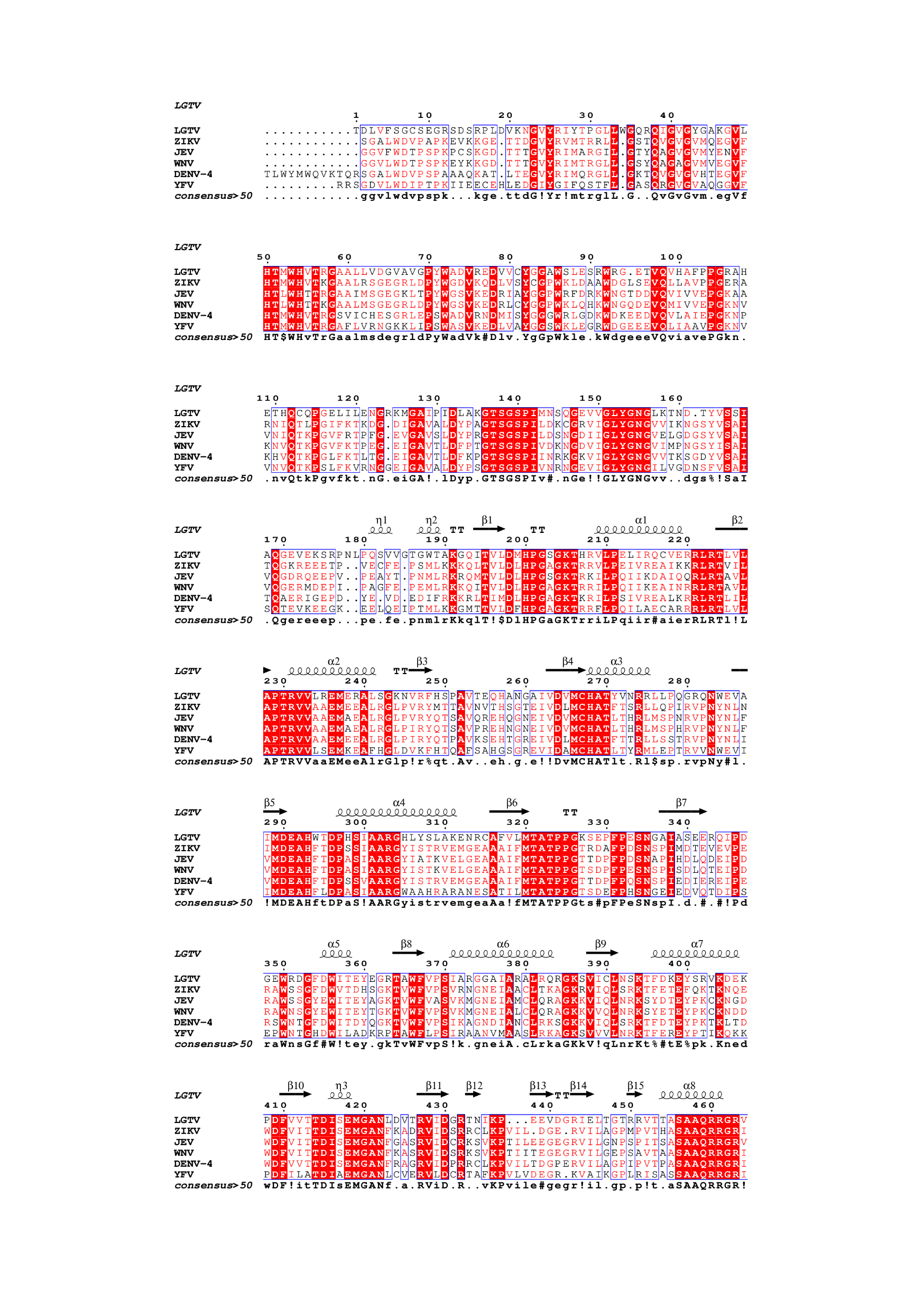


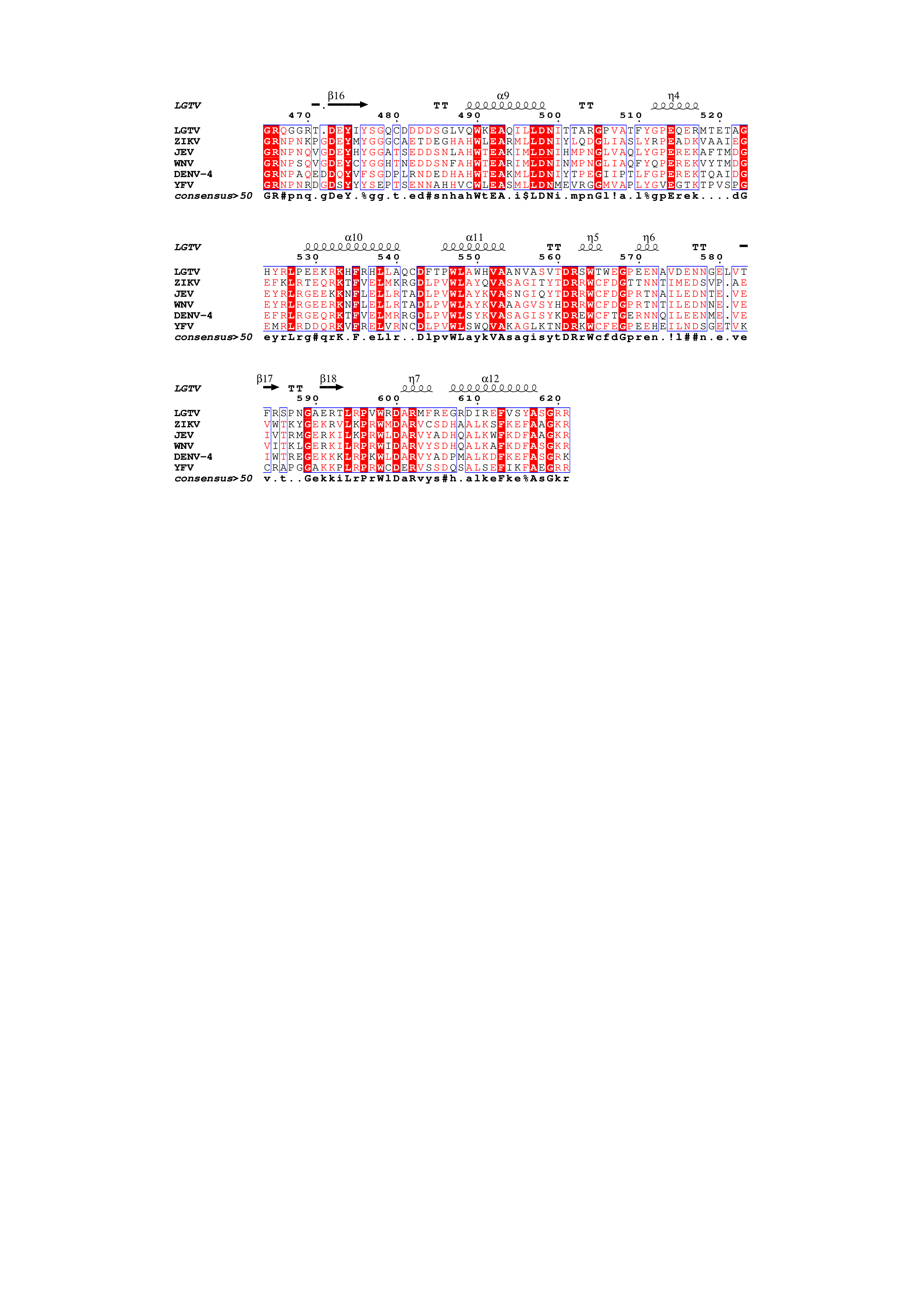


**Fig. S1.** Structure-based alignment of Flaviviridae helicases.

**Table S1.** Comparison of residues contacting with RNA from Langat virus, ZIKV and DENV4 helicase.


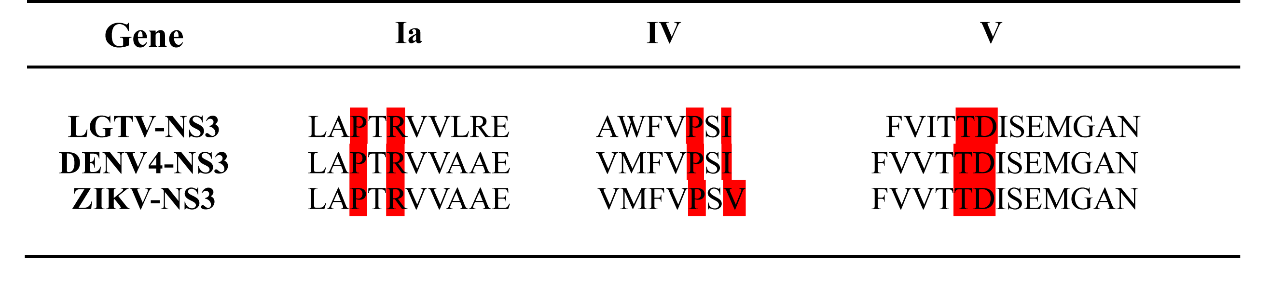


**Table S2.** The ATPase activity comparison of different flaviviruses helicase.

| **Flavivirus** | **K_m_ (mM)** | **K_cat_ (S^−1^)** | **K_cat_/K_m_ (M^−1^·S^−1^)** |
| --- | --- | --- | --- |
| LGTV | 0.336±0.053 | 0.096±0.004 | 285.31 |
| ZIKV | 0.285±0.069 | 2.432±0.250 | 8533.33 |
| ILHV | 0.317±0.036 | 1.088±0.068 | 3432.18 |
| MVEV | 0.380±0.030 | 5.300±0.024 | 13947.37 |
| DENV4 | 0.300±0.223 | 5.800±0.213 | 19333.33 |

**Table S3.** Candidate inhibitors of LGTV helicase by virtual screening.

| **Compound** | **Score (kcal/mol)** | **Cas** | **Formula** | **MW** | **Targets** |
| --- | --- | --- | --- | --- | --- |
| Zafirlukast | -13.31 | 107753-78-6 | C_31_H_33_N_3_O_6_S | 575.68 | leukotriene D4 (LTD4) receptor |
| Bananins | -11.28 | 665026-57-3 | C_14_H_17_NO_8_ | 327.29 | ATPase |
| Hypericin | -10.84 | 548-04-9 | C_30_H_16_O_8_ | 504.44 | PKC, MAO, dopamine-beta-hydroxylase, reverse transcriptase, telomerase and CYP |
| Sennoside A | -10.05 | 81-27-6 | C_42_H_38_O_20_ | 862.74 | HIV-1 RDDP, ribonuclease H |
| RK-33 | -9.11 | 1070773-09-9 | C_23_H_20_N_6_O_3_ | 428.44 | DDX3 |
| SSYA10-001 | -8.72 | 675104-49-1 | C_12_H_12_N_4_O_2_S_2_ | 308.38 | human papillomavirus E6 |
| PF-03715455 | -8.72 | 1056164-52-3 | C_35_H_34_ClN_7_O_3_S_2_ | 700.27 | p38 MAPK |
| Divanchrobactin | -8.52 | 1233718-16-5 | C_32_H_44_N_10_O_13_ | 776.76 | ZIKV helicase |
| Quercitrin | -7.23 | 522-12-3 | C_21_H_20_O_11_ | 448.38 | Quercetin 3-rhamnoside  Ribosomal S6 Kinase (RSK), Oxygen Species (ROS) |
| Citicoline sodium | -7.14 | 33818-15-4 | C_14_H_25_N_4_NaO_11_P_2_ | 510.31 | Reactive Oxygen Species (ROS), Caspase |


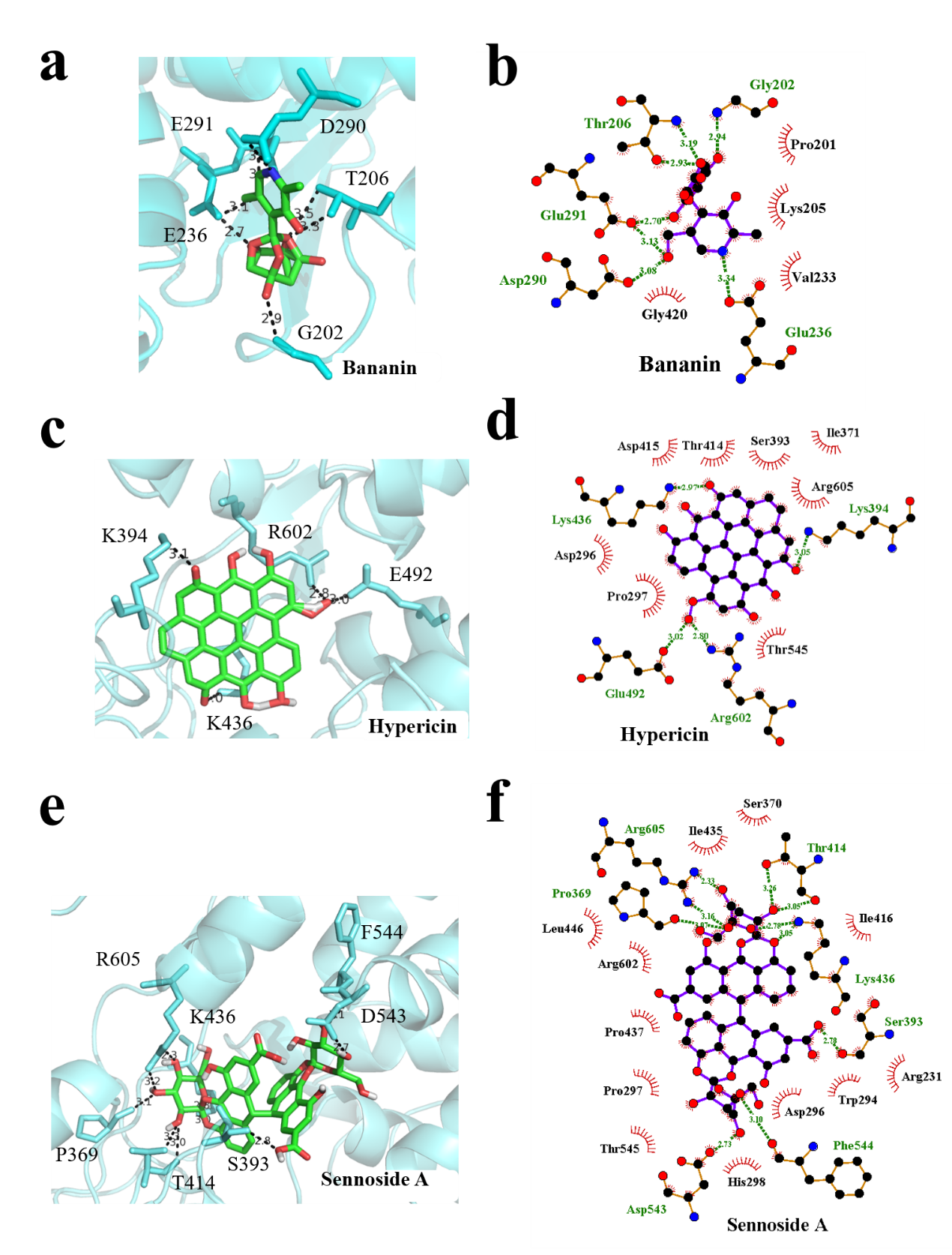


**Fig. S2.**Conserved binding mode of different inhibitors across Langat virus helicase. **a-b**, Detailed view of Bananin binding in NTP binding pocket of Langat virus helicase. **c-d**, Detailed view of Hypericin binding in NTP binding pocket of Langat virus helicase. **e-f**, Detailed view of Sennoside A binding in NTP binding pocket of Langat virus helicase.


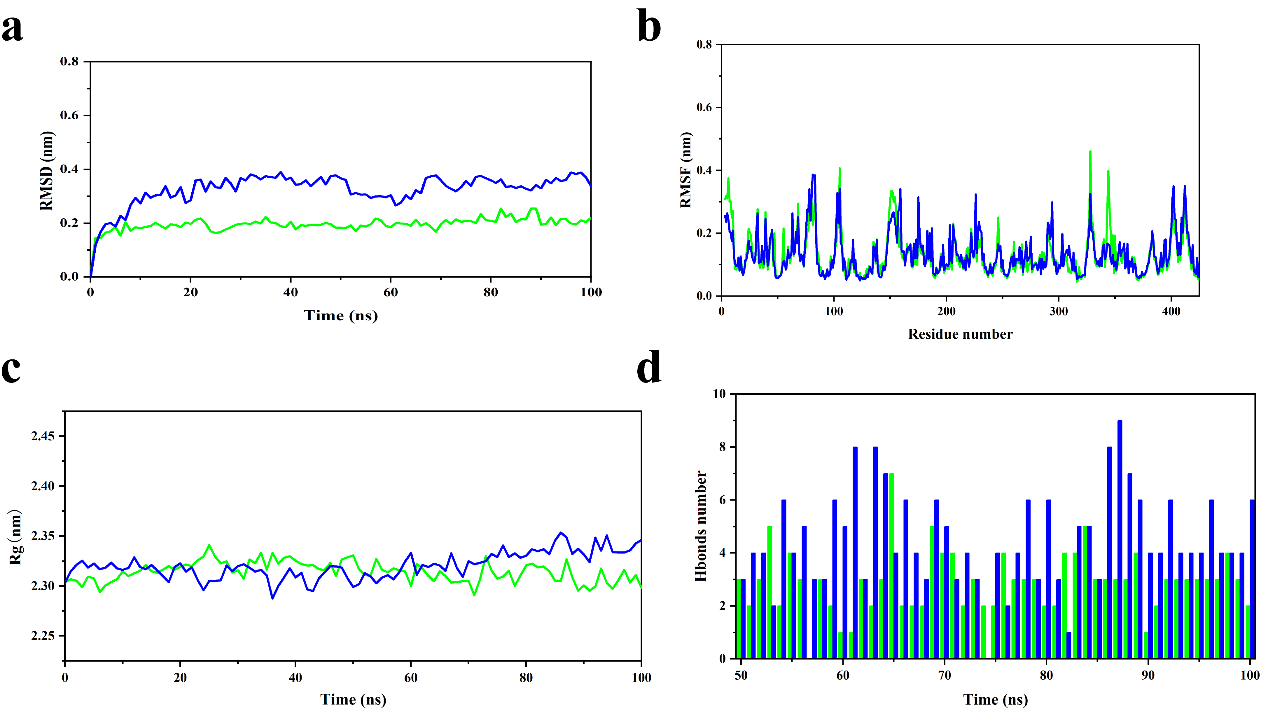


**Fig. S3.**Discovery of inhibitors targeting Langat virus helicase. **a-b,** RMSD and RMSF plot during molecular dynamics simulations of Langat helicase with Hypericin (blue) and Sennoside A (green). **c-d,** The Rg and hydrogen bond number of Langat helicase with Hypericin (blue) and Sennoside A (green).


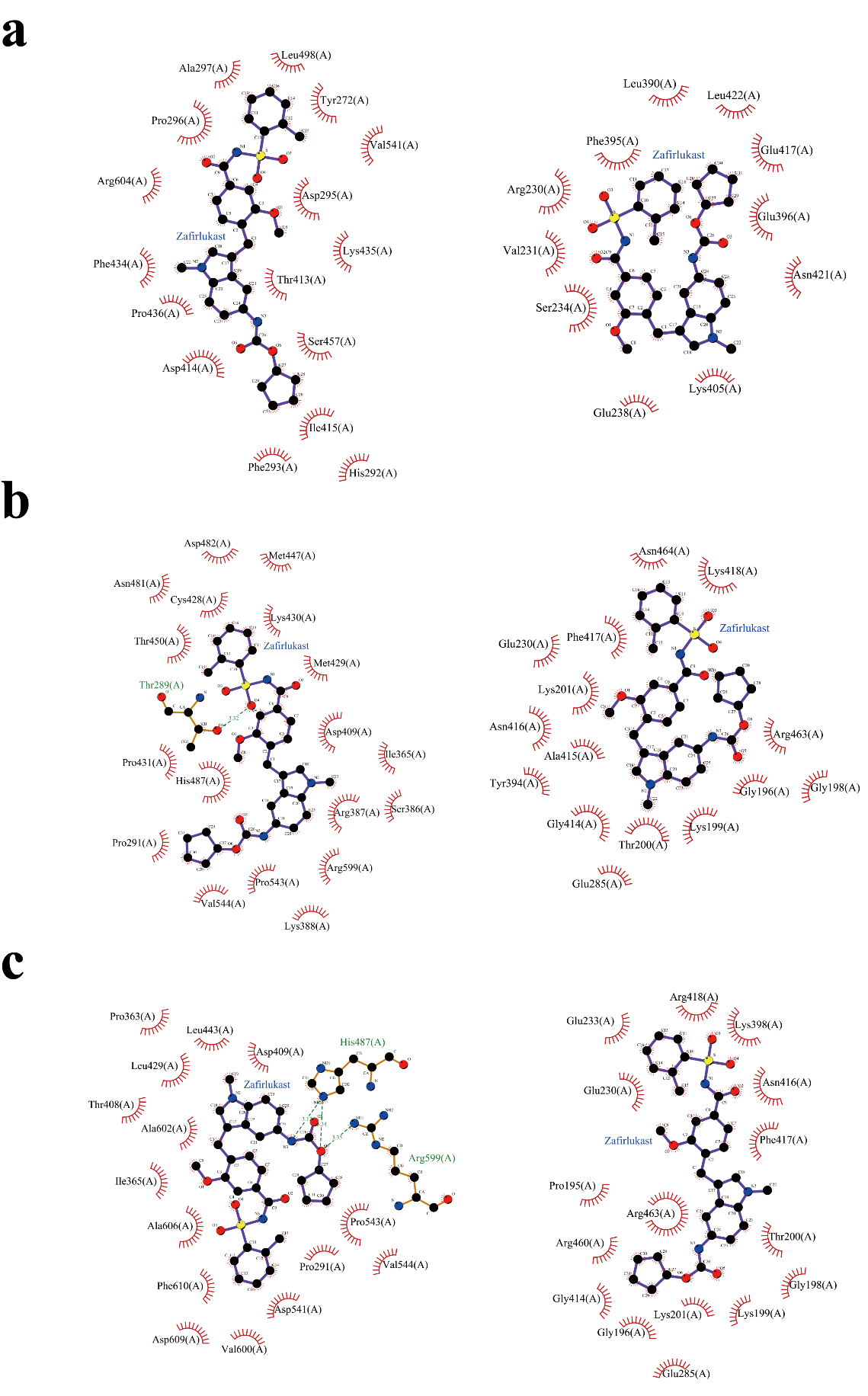


**Fig. S4.**Conserved binding mode of Zafirlukast across flavivirus helicases. **a-c**, Detailed view of Zafirlukast binding in both RNA binding groove (left) and NTP binding pocket (right) of YFV(a), DENV2(b), DENV4(c).


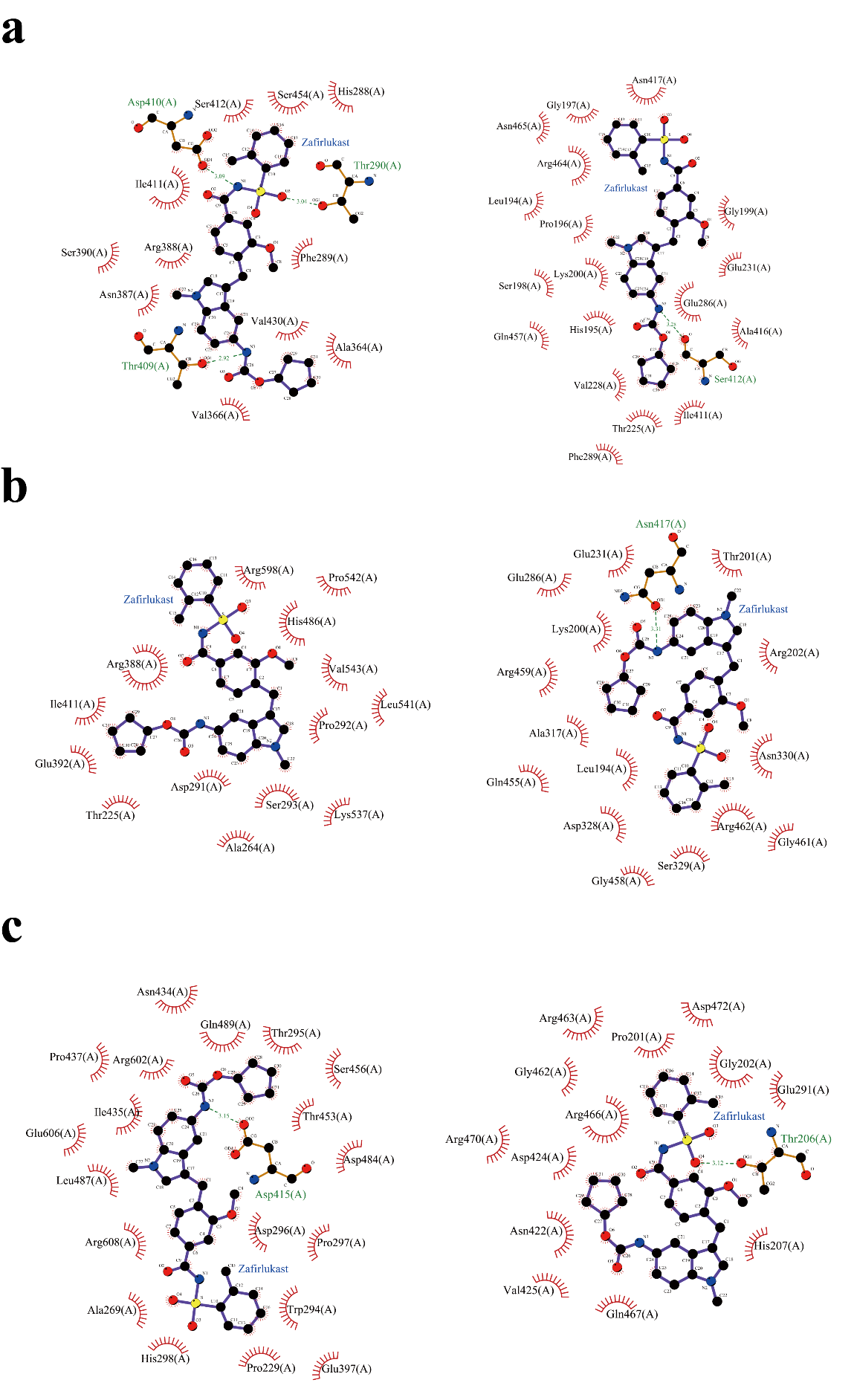


**Fig. S5.**Conserved binding mode of Zafirlukast across flavivirus helicases. **a-c**, Detailed view of Zafirlukast binding in both RNA binding groove (left) and NTP binding pocket (right) of JEV(a), ZIKV(b) and TBEV(c) helicase.
